# Optimization of carbon ion and proton treatment plans using the raster-scanning technique for patients with unresectable pancreatic cancer

**DOI:** 10.1186/s13014-015-0538-x

**Published:** 2015-11-21

**Authors:** Constantin Dreher, Daniel Habermehl, Swantje Ecker, Stephan Brons, Rami El-Shafie, Oliver Jäkel, Jürgen Debus, Stephanie E. Combs

**Affiliations:** Department of Radiation Oncology, University Hospital of Heidelberg, INF 400, 69120 Heidelberg, Germany; Department of Radiooncology, Klinikum rechts der Isar, Ismaninger Str. 22, 81675 Munich, Germany; Heidelberg Ion Beam Therapy Center (HIT), INF 450, 69120 Heidelberg, Germany; Department of Medical Physics in Radiation Oncology, German Cancer Research Center, INF 280, 69120 Heidelberg, Germany

**Keywords:** Locally advanced pancreatic cancer, LAPC, Carbon ion, Proton, HIT

## Abstract

**Background:**

The aim of the thesis is to improve radiation plans of patients with locally advanced, unresectable pancreatic cancer by using carbon ion and proton beams.

**Patients and methods:**

Using the treatment planning system *Syngo RT Planning* (Siemens, Erlangen, Germany) a total of 50 treatment plans have been created for five patients with the dose schedule 15 × 3 Gy(RBE). With reference to the anatomy, five field configurations were considered to be relevant. The plans were analyzed with respect to dose distribution and individual anatomy, and compared using a customized index.

**Results:**

Within the index the three-field configurations yielded the best results, though with a high variety of score points (field setup 5, carbon ion: median 74 (range 48–101)). The maximum dose in the myelon is low (e.g. case 3, carbon ion: 21.5 Gy(RBE)). A single posterior field generally spares the organs at risk, but the maximum dose in the myelon is high (e.g. case 3, carbon ion: 32.9 Gy(RBE)). Two oblique posterior fields resulted in acceptable maximum doses in the myelon (e.g. case 3, carbon ion: 26.9 Gy(RBE)). The single-field configuration and the two oblique posterior fields had a small score dispersion (carbon ion: median 66 and 58 (range 62–72 and 40–69)). In cases with topographic proximity of the organs at risk to the target volume, the single-field configuration scored as well as the three-field configurations.

**Conclusion:**

In summary, the three-field configurations showed the best dose distributions. A single posterior field seems to be robust and beneficial in case of difficult topographical conditions and topographical proximity of organs at risk to the target volume. A setup with two oblique posterior fields is a reasonable compromise between three-field and single-field configurations.

## Background

Pancreatic cancer patients are still having a dismal prognosis. About 277,000 people worldwide die each year due to this disease [1]. To date resection is considered to be the only curative treatment. In case of locally advanced unresectable pancreatic cancer (LAPC), neoadjuvant treatment approaches including combined chemoradiation with gemcitabine have proven efficacy towards tumor downsizing and lead to a secondary resectability in approximately 30 %, even in case of local relapse after primary surgery [2–6]. Modern radiotherapy techniques including IMRT (Intensity-Modulated Radiotherapy) and IGRT (Image-Guided Radiotherapy) offer more conformal dose distributions and thus dose to organs at risk (OAR) can be reduced significantly. Recently it was shown that IMRT treatment in pancreatic cancer patients can provide comparable or less gastrointestinal toxicity than by conventional radiotherapy and therefore contributes to the clinical benefit of the treated patients, especially when used as dose-escalation treatment in combination with IGRT [7, 8]. Charged particle therapy represents an emerging technological advance in oncology. Particle therapy is characterized by an inverted depth-dose-curve, which leads to a low dose deposition within the entry channel and a well-defined high local dose deposition in the Bragg Peak region [9]. The so called Spread Out Bragg Peak (SOBP) allows to irradiate precisely, sparing surrounding normal tissue – both carbon ion and proton radiotherapy have highly conformal dose distributions with high dose deposit in the target volume and an increased sparing of the OARs [9]. High-LET (linear energy transfer) carbon ion beams are characterized by high dose deposition in their trajectory. This results in a high amount of clustered double-strand breaks in the cells‘DNA (Deoxyribonucleic acid). So, in contrast to photon and proton beams, carbon ion beams cause an enhanced reduction in clonogenic survival of pancreatic and also of hepatic cell lines [10–13]. Carbon ions in particular offer a higher biological effectiveness due to enhanced and prolonged DNA damaging and induction of bulky lesions, which can be translated into higher RBE (Relative Biological Effectiveness) values [11, 13, 14]. Particle beams are notably appropriate in hepatobiliary and pancreatic malignancies, where radiosensitive normal tissues (e.g. liver, kidneys) are surrounding the target volume [15–17].

There are also encouraging clinical results from Japanese particle therapy facilities, that have conducted small clinical trials and gained experience with carbon ion treatment, using different treatment protocols over the last few years [16, 18]. Nevertheless, particle therapy of abdominal organs is very complex. Dose application has to be analyzed carefully. That’s the reason why the purpose of this study is to evaluate different plan optimization strategies as a preparation for the clinical practice using active raster scanning technology [19]. Different proton and carbon ion field configurations are analyzed with regard to dose distribution and individual anatomy, using a customized rating scheme.

## Patients and methods

### Patient characteristics and anatomy criteria

The medical ethics commission of the medical faculty of Heidelberg consented to this in silico study (S-483/2011). Five patients with locally advanced, unresectable pancreatic cancer were included in this study. They were randomly selected from patients treated with standard photon plans at our institution. For treatment planning CT (computed tomography) scans were performed with and without contrast agent and under free breathing. Patients were immobilized in supine position.

Over the cranio-caudal direction of the target volume the minimal distance between two structures was measured in each horizontal slice, and afterwards the mean value was calculated. The mean value depends on variations due to shape and orientation of two structures over the total target volume extension – the following measures were calculated:

1. Mean Xmin kidney ri-le = mean minimum distance between both kidneys
2. Mean Xmin target-kidney le = mean minimum distance between target and left kidney
3. Mean Xmin target-kidney ri = mean minimum distance between target and right kidney
4. OAR intersection = intersection between target and OARs

Patient characteristics and anatomical criteria are summarized in “Table 1”.

**Table 1:** Patient characteristics and anatomy criteria

Patient characteristics:	Case1	Case2	Case3	Case4	Case5
Gender	Male	Male	Female	Male	Male
Age at CT scan (years)	71	77	64	67	67
Location	Caput	Caput	Caput	Caput/Corpus	Caput/Corpus
Target volume (cm^3^)	332.63	92.31	165.75	224.39	150.35
Anatomical characteristics:					
Mean Xmin kidney ri-le (cm)	10.1	9.6	7.5	10.6	7.5
Mean Xmin target-kidney le (cm)	5.4	6.0	3.1	5.3	4.1
Mean Xmin target-kidney ri (cm)	3.2	3.2	3.6	2.5	2.5
OAR-Intersection	Large intestine	Large intestine	Large intestine, Stomach/duo-denum, Liver	Large intestine	Large intestine, Stomach/duo-denum, Liver

### Target volume definition

The treatment planning CT scans with the patients’original volumes of photon irradiation have been transferred to our ion beam treatment planning system. The original target was made up of a PTV (planning target volume) including elective nodal irradiation and a boost volume including the GTV (gross tumor volume) and a margin of 2–4 mm at the discretion of the responsible specialist. The boost volumes are defined as the target volumes in the presented cases.

### Treatment Planning System (TPS)

Treatment planning was performed for particle beams using the raster-scanning technique [19]. Treatment planning computation was done by TPS *Syngo RT Planning* (Siemens, Erlangen, Germany), using the effective dose calculation model as described by Krämer & Scholz (Local Effect Model, LEM) [20]. Treatment planning with proton beams assumes a fixed RBE value of 1.1. Planning is possible by the use of *single field uniform dose optimization* (SBO, Single Beam Optimization) or *multiple field optimization* (IMPT, Intensity Modulated Particle Therapy). Both tools are using intensity modulation, but SBO allows relative weighting factors for each beam. These beams are optimized independently and add up to 100 % of the prescribed dose. IMPT integrates all beams and optimizes simultaneously.

### Dose prescription

At our institution a slightly hypofractioned dose regime has been established for carbon ion irradiation, with a single dose of 3 Gy(RBE) as described in our forthcoming clinical trial on dose escalated carbon ion therapy for patients with pancreatic cancer [21]. We chose a fraction number of 15, representing the second escalation dose in the above mentioned PHOENIX trial. The calculated total dose adjusted to the fractionation effect according to the linear-quadratic model and an α/β-ratio of 2 Gy would result in approximately 56 Gy(RBE) (BED 2 Gy(RBE)). This dose remains to our opinion realistic and is effective for both neoadjuvant and definitive treatment.

### Field Setup (FS)

Five different FS at the gantry were considered to be relevant for this study (Table 2). Field configurations including three fields use the SBO tool - to minimize dosimetric uncertainty due to putative anatomical variations the greatest weight was given to the posterior field [22]. These five FS were used for all cases – though slightly adapted to different topography. The gantry beam angles are described according to the International Electrotechnical Commission (IEC).

**Table 2:**
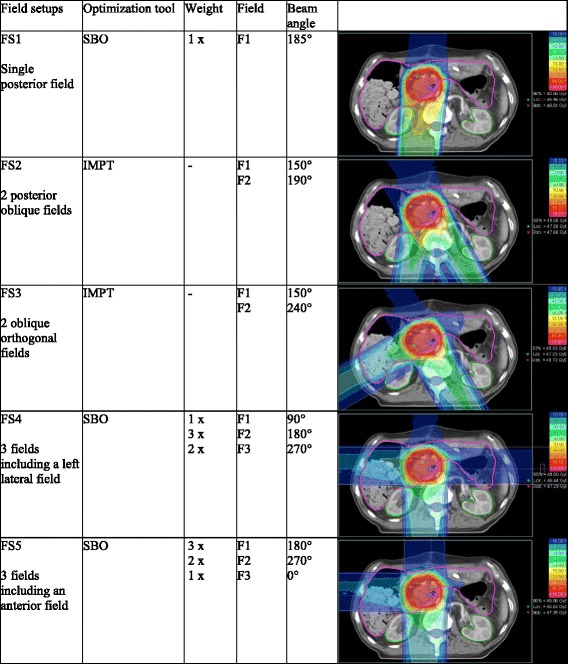
Field setups: characteristics

### Treatment plans

A total of 50 treatment plans were created – taking into account 5 FS, both for carbon ions and protons. The constraints for optimization were put on the same level. For treatment evaluation the original structure sets of photon irradiation have been integrated in our TPS, which is why the OAR *duodenum* is not available, but integrated in the OAR *stomach/dd*.

The criteria and the index for comparison are summarized in “Table 3”. In general, the DVH (Dose-Volume-Histogram)-parameters were geared to those constraints, which are significant for toxicity and derived from daily clinical practice. As critical tolerance doses were not exceeded, we decided to take lower tolerance limits to better distinguish the different plans. The OAR criteria of the myelon, the kidneys and the liver are of equal value; stomach/dd and large intestine 33 % less; skin 66 % less. So, in comparison to stomach/dd etc., the criteria value of myelon, kidneys and liver is increased. This distinction is due to the field configurations, for which these organs are consequently exposed to higher doses. The plans were compared with each other by using a specific customized score - the index allocates points amongst the different plans as follows:

**Table 3:** Target and OAR criteria

Structure	Constraint	Maximum points	Sum	Score	
Target:	V44 ≥ 95 %	10	40	Target-Criteria	Cumulative-Criteria
	1-V42.75 < 1 %	10			
	Max < 48.15 Gy(RBE)	10			
	Min > 40.00 Gy(RBE)	10			
Myelon:	Max < 24.00 Gy(RBE)	15	15	OAR-Criteria	
Each Kidney:	V15 < 15.00 %	5	15		
	D25 < 10.00 Gy(RBE)	5			
	Mean < 12.00 Gy(RBE)	5			
Liver:	V20 < 12.50 %	5	15		
	V10 < 20.00 %	5			
	Mean < 10.00 Gy(RBE)	5			
Stomach/DD:	Max < 20.00 Gy(RBE)	5	10		
	V20 < 15.00 %	5			
Large intestine:	Max < 20.00 Gy(RBE)	5	10		
	V35 < 10.00 %	5			
Skin:	Max Isodose < 50.00 %	5	5		

Five FS were compared per case and per radiation modality (carbon ions, protons). If a plan does not meet the criteria, it does not receive any points for this criterion. Among the plans meeting the criteria, the plan achieving the best value receives the maximum number of points. The other plans receive fewer points in linear intervals (point grading of the only myelon criterion: 15–12–9–6–3). The cumulative criteria are made up of both target criteria and OAR criteria. The target criteria represent 32 % of the score. The OAR criteria get the remaining 68 % - the relative distribution is identical to the OAR criteria.

## Results

### DVH-parameters for proton treatment plans

**Fig. 1:**
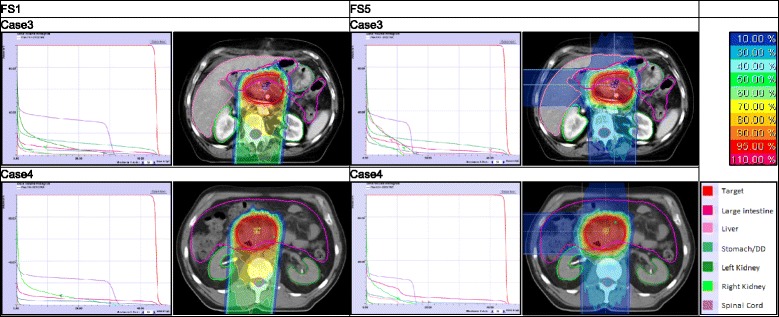
DVH (Dose-Volume-Histogram) for case 3 and 4 with FS (field setup) 1 and 5: proton

FS 5 (Fig. 1) has a high dose coverage of the target with V44 = 98.7 % and 98.6 % for case 3 and 4. The maximum dose (D_max_) in the myelon, and dose criteria for both kidneys are met in both cases. DVH-parameters with FS 4 are similar to those with FS 5.

With regard to FS 2 and 3, the target criteria are satisfied in both cases. D_max_ in the myelon is satisfied in case 3, but narrowly missed in case 4 by both FS (FS 2 and 3: 25.2 Gy(RBE) and 27.3 Gy(RBE)). In case 3 and 4 FS 3 leads to an increased dose deposition in both kidneys. In contrast to that, FS 2 is fulfilling the criteria.

FS 1 (Fig. 1) reaches a high V44 in the target: 97.5 % and 98.1 % (case 3 and 4). The dose criterion for the myelon cannot be met (case 3 and 4: 32.4 Gy(RBE) and 34.0 Gy(RBE)), but those for both kidneys can. The mean dose (D_mean_) in the right kidney is only half of the dose with FS 5.

### DVH-parameters for carbon ion treatment plans (Fig. 2)

**Fig. 2:**
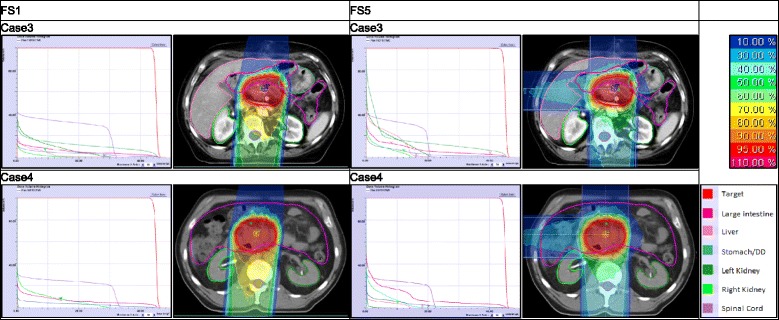
DVH (Dose-Volume-Histogram) for case 3 and 4 with FS (field setup) 1 and 5: carbon ion

FS 5 (Fig. 2) meets the target criterion V44: 95.8 and 96.2 % for case 3 and 4. D_max_ in the myelon is 21.5 Gy(RBE) and 23.2 Gy(RBE) in case 3 and 4. For both cases, the kidney criteria are satisfied. DVH-parameters of FS 4 are similar to those of FS 5 for both cases.

In case 3 FS 3 does not meet the target criterion and the kidney criteria - but it does in case 4. With regard to D_max_ in the myelon, it is the other way around. FS 2 meets with the target criterion V44 and kidney criteria in both cases (but for the D_max_). D_max_ in the myelon is 24.4 Gy(RBE) and 27 Gy(RBE) in case 4 and 3.

FS 1 (Fig. 2) meets the target criterion V44 for both cases (96.8 and 95.1 % in case 3 and 4). For case 3 the target’s volume dose is higher than the one with FS 5. D_max_ in the myelon is 32.9 Gy(RBE) and 34 Gy(RBE) in case 3 and 4. DVH-parameters in the kidneys (D_mean_ (right and left kidney) = 0.4 Gy(RBE) and 2.9 Gy(RBE)) are low in case 3. The same is true for case 4.

### Index evaluation (Fig. 3)

**Fig. 3:**
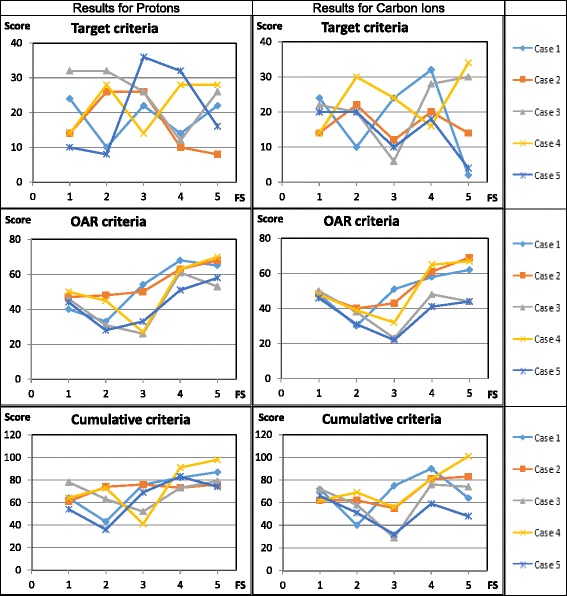
Index results for proton and carbon ion treatment plans

Protons: FS 4 and 5 yielded the highest scores for the OAR criteria. For case 3 FS 1 achieved the same score as the three-field configurations. Carbon ions: FS 5 has a wide score dispersion for the target criteria (Median 14 (range 2–34)) and cumulative criteria (Median 74 (range 48–101)). Regarding OAR criteria, FS 4 and 5 achieved the highest median scores (58 and 62). Nevertheless, score dispersion is the smallest for FS 1 (range 46–50). FS 2 also has a very small score dispersion (range 30–40). On top of that, in case 3 FS 1 gets almost the same score as the three-field configurations, in case 5 FS 1 even exceeds their score points.

Evaluating the anatomical criteria (Table 1), the cases 3 and 5 clearly offer a topographic proximity of the target to the OARs and the OARs to each other. The mean distance between the right and the left kidney, and the mean distance between the kidneys and the target itself are small in these cases.

## Discussion

Our study focused on treatment planning strategies in patients with unresectable pancreatic cancer. 50 treatment plans for proton and carbon ion beams were evaluated. We developed a score taking into account target volume coverage and OAR parameters to provide a basis for decision making during the planning process for this patient group. The score demonstrates, that for target volume coverage, all beam angles and combinations provide nearly equivalent scores with a slight advantage for multiple field plans.

In summary, a three-field setup achieved consistently high values throughout the cases. A one-field arrangement with a single posterior field showed in some cases comparable results and overall very little score dispersion. Nevertheless, the maximum doses in the myelon were thoroughly high. But this field configuration seems to be of advantage in cases of topographic proximity of radiosensitive OARs to each other and to the target volume. The field-setup with two posterior oblique fields through the kidneys showed satisfying results, was able to spare the myelon, and score dispersion was quite small as well.

Particle therapy of abdominal organs is very complex, as inter- and intraindividual changes in organ motion and bowel gas movement may have a serious impact on ion beam dosimetry [22, 23]. Kumagai and colleagues reported a treatment plan analysis of passive scattered carbon ion beams at their facility and found out, that both anterior-posterior and left-right field constellations caused the highest dose affections (mainly because of gastrointestinal gas bubbles) [22]. The three-field configurations include such fields, which is why they have to be evaluated critically. Our results also point to more robustness of a single posterior field compared to three-field arrangements. In this setting beam paths are practically not affected by gas fillings in stomach (left-right) and intestine (anterior-posterior). On top of that, inter- and intrafractional variations due to breathing may cause relevant changes in the beam path leading to adverse effects. Taniguchi and colleagues further analyzed doses in duodenum and stomach in patients with unresectable pancreatic carcinoma treated with a five-fraction protocol. Results show a decreasing dose to the OARs during expiration compared to inspiration [24]. The interaction of beam and organ motion can cause interplay effects and potentially lead to (unexpected) dose variations in target structures and thus unwanted normal tissue exposure [25]. However, intra- and inter-fractional changes are described but not totally understood, which is why we need re-planning scenarios, especially in scanned ion beam treatment, where slight changes may result in significant dose variations [22–24, 26]. In general, we are referring to Japanese experiences in ion beam therapy of LAPC. But one has to be clear about the fact, that irradiation was performed by the use of scattered ion beam therapy. Our department made investigations in robustness in scanned ion beam therapy, which we can rely on. Batista et al. has presented data about pancreatic cancer, that supported our results. A single posterior field and two oblique posterior fields show good results in case of robustness [27]. On top of that Richter et al. has made investigations in the topic of robustness of ion beam irradiation of liver tumors. The group was able to show, that fractionation is a potential tool to reduce dose inhomogenity by interplay effects [28, 29]. That is one of the reasons why our PHOENIX trial has dose escalation steps starting with 12 fractions [21].

In out treatment plan computations doses to OARs were thoroughly uncritical. But distinct field setups lead to different risk profiles. Whereas posterior fields deposit higher doses to the myelon and partial volumes of the kidneys, right lateral fields are surely affecting partial liver volumes. Higher doses in intestinal structures were found when left lateral and anterior fields were used (three-field configurations). Even though only small sub-volumes received remarkable doses, this could theoretically result in clinically relevant complications. The experience on comparable dose protocols with intestinal structures in close proximity to the target volume is based on carbon ion therapy of sacral chordoma and locally recurrent rectal cancer, where no higher gastrointestinal toxicities were recorded [30, 31].

The single posterior field setup is of major concern, because of a steep RBE-increase at the distal end of the SOBP, which could result in unexpected high doses to the small intestine ventrally of the pancreatic tumor. Calculated doses to intestines were in the range of tolerance. With regard to the DVH, the single posterior field can spare most of the OARs. High doses in the myelon were calculated, but did not exceed general QUANTEC (Quantitative Analyses of Normal Tissue Effects in the Clinic) dose constraints [32].

Nevertheless, tolerance doses of the OARs are not well defined for carbon ion irradiation and are extrapolated from photon-based data as long as no new dose-volume thresholds are defined. An important limitation of the LEM-based TPS is also the underlying biological dataset. According to our current clinical practice we do not differentiate the radiobiological characteristics of normal tissue and tumor during treatment plan optimization. Experience with carbon ion beams based on newer biological optimized LEM-algorithms within the TPS are only theoretical and not debated in this work [33].

Topographical influence and advantage of the single-field configuration is mainly expressed by carbon ion beams. It is likely that the proton beams are at a disadvantage due to their wider lateral dose gradient [9]. Due to this beam broadening, especially with deeper located targets, smearing of the dose distribution possibly results in plans inferior to advanced photons. Basically, a direct comparison between carbon ion and proton beams could be drawn but this may not be useful, as we are currently still working with an earlier version of LEM [34]. Therefore the dose in the proximal part of the beam channel is rather overestimated and the dose in the target itself is underestimated as recently demonstrated by Grun and co-workers with an improved version of the local effect model (LEM IV) [35]. Carbon ion radiation would result in more beneficial dose distributions both in the target and OARs than shown in the presented data.

Nonetheless, our study was able to present possible field setups and evaluated them by the use of a customized score. With reference to our results, particle therapy of pancreatic cancer is possible by the use of all 5 field setups. Distinct topographical conditions should be taken into account.

Future work of our group will focus on even more differentiated radiobiological-based treatment planning, which includes different α/β ratios for normal and target tissues.

## Conclusion

In summary, field configurations with three fields showed the best dose distributions – for both carbon ion beams and proton beams. Nevertheless, three-field configurations are highly influenced by gastrointestinal variations. A single posterior field deposits high doses in the myelon, but seems to be the most robust one and showed good results for difficult and varying topographical conditions. A setup with two oblique posterior fields generated reproducible results and can be set as a reasonable compromise between three fields and one field configurations.

## Abbreviations

BED: biologically effective dose
CT: computed tomography
DEGRO: German Radiation Oncology Society
D_max_: maximum dose
D_mean_: mean dose
DNA: Deoxyribonucleic Acid
DVH: Dose-Volume-Histogram
FS: Field Setup
GTV: gross tumor volume
HIT: Heidelberg Ion Beam Therapy Center
IEC: International Electrotechnical Commission
IGRT: Image-Guided Radiotherapy
IMPT: Intensity Modulated Particle Therapy
IMRT: Intensity-Modulated Radiotherapy
LAPC: locally advanced pancreatic cancer
LEM: Local Effect Model [20]
LET: Linear Energy Transfer
OAR: organ at risk
PTV: planning target volume
QUANTEC: Quantitative Analyses of Normal Tissue Effects in the Clinic
RBE: Relative Biological Effectiveness
SBO: Single Beam Optimization
SOBP: Spread Out Bragg Peak
TPS: Treatment Planning System
